# Proton dynamics in cancer

**DOI:** 10.1186/1479-5876-8-57

**Published:** 2010-06-15

**Authors:** Veronica Huber, Angelo De Milito, Salvador Harguindey, Stephan J Reshkin, Miriam L Wahl, Cyril Rauch, Antonio Chiesi, Jacques Pouysségur, Robert A Gatenby, Licia Rivoltini, Stefano Fais

**Affiliations:** 1 Unit of Immunotherapy of Human Tumors, Fondazione IRCCS Istituto Nazionale Tumori, Milan, Italy; 2 Department of Therapeutic Research and Medicines Evaluation, Unit of Antitumor Drugs, Istituto Superiore di Sanità, Rome, Italy; 3 Institute for Clinical Biology and Metabolism, Vitoria, Spain; 4 Department of General and Environmental Physiology, University of Bari, Bari, Italy; 5 Department of Cell Biology, Johns Hopkins, Baltimore, MD, USA; 6 School of Veterinary Medicine & Science, University of Nottingham, College Road, Sutton Bonington, LE12 5RD, UK; 7 Institute of Developmental Biology & Cancer, CNRS, Centre A. Lacassagne, University of Nice, France; 8 Department of Radiology and Integrative Mathematical Oncology, Moffitt Cancer Center, Tampa, FL, USA; 9 Cancer Center Karolinska, Karolinska Institute, Stockholm, Sweden

## Abstract

Cancer remains a leading cause of death in the world today. Despite decades of research to identify novel therapeutic approaches, durable regressions of metastatic disease are still scanty and survival benefits often negligible. While the current strategy is mostly converging on target-therapies aimed at selectively affecting altered molecular pathways in tumor cells, evidences are in parallel pointing to cell metabolism as a potential Achilles' heel of cancer, to be disrupted for achieving therapeutic benefit. Critical differences in the metabolism of tumor versus normal cells, which include abnormal glycolysis, high lactic acid production, protons accumulation and reversed intra-extracellular pH gradients, make tumor site a hostile microenvironment where only cancer cells can proliferate and survive. Inhibiting these pathways by blocking proton pumps and transporters may deprive cancer cells of a key mechanism of detoxification and thus represent a novel strategy for a pleiotropic and multifaceted suppression of cancer cell growth.

Research groups scattered all over the world have recently started to investigate various aspects of proton dynamics in cancer cells with quite encouraging preliminary results. The intent of unifying investigators involved in this research line led to the formation of the "International Society for Proton Dynamics in Cancer" (ISPDC) in January 2010. This is the manifesto of the newly formed society where both basic and clinical investigators are called to foster translational research and stimulate interdisciplinary collaboration for the development of more specific and less toxic therapeutic strategies based on proton dynamics in tumor cell biology.

## 

Cancer remains a leading cause of death in the world today. Despite decades of research to identify novel therapeutic approaches, durable regressions remain rare in patients with advanced disease, and survival benefits from new therapies are often negligible. Early diagnosis and treatment, rather than successful therapeutic intervention late in the disease process, are mostly responsible for the decreased mortality observed in some types of cancer (WHO).

The war against cancer has been heavily influenced by the principle of Paul Ehrlich's 'magic bullet', an idea originally introduced more than 100 years ago, and validated by the discovery of antibiotics 50 years later. Clinical cancer therapy implicitly assumes that similar magic bullets for cancer can be found with sufficient diligence. However, all attempts to find new drugs that selectively target and kill tumor cells have not been successful [1] and today we are still waiting for the magic bullet directed against most malignant tumors. As a result, alternative therapeutic approaches aimed at controlling rather than curing this disease are increasingly advocated [2].

In the past decade cancer imaging with FdG PET has become commonplace demonstrating that more than 90% of clinical cancers of sufficient size to be imaged take up glucose several fold more than adjacent normal tissue. Remarkably, although this appears to be the single most common property of the "tumor phenotype" the causes and consequences of glycolysis have rarely been considered as a therapeutic target. We propose that a new approach towards the war against cancer can be found in re-considering the critical differences in the metabolism of a tumor cell as compared to a normal one. More than 80 years ago the Nobel Prize winner Otto Heinrich Warburg discovered that tumors use glycolytic metabolism even in the presence of normal oxygen tension, an apparent paradox, since glycolysis is 18-fold less efficient than oxidative phosphorylation in producing energy (ATP). He also found that cancer cells were, unlike normal cells, able to live in the acidic environment that developed as a consequence of elevated lactic acid production by glycolysis. Today, we know that the metabolic changes occurring during cancer progression are mediated by two major pathways, i.e. the activity of hypoxia-inducible factor 1 (HIF-1) and oncogene activation [3,4]. The selective ability of tumor cells to adapt their metabolism to a hostile microenvironment is revealing new therapeutic targets to selectively eliminate cancer cells [5,6].

We propose that the development of new anti-cancer therapies should include a focus on understanding both abnormal glucose metabolism and mechanisms used by cancer cells to survive and proliferate in the hostile microenvironment. In the latter, a dominant role is played by protons accumulating at the tumor site as a result of highly proliferating cancer cells relying on glycolysis with high lactic acid production and limited removal due to poor perfusion. To avoid acidification of intracellular pH, glycolytically-produced acid must be extruded by tumor cells through a number of proton transporters, such as V-ATPase [7], the Na^+^/H^+ ^exchanger (NHE) [8], the carbonic anhydrases [9], the proton linked monocarboxylate transporter MCTs [4], the Cl^-^/HCO3^- ^exchangers and ATP synthase [10]. The increased activity of these transporters cause reversal of the normal intra-extracellular pH gradients, so that cancer cells produce significant acidification of the extracellular microenvironment, while they maintain a normal or slightly alkaline pH.

In contrast to cancer cells, the acidic extracellular space reduces viability and function of most normal cells including cytotoxic T cells that ordinarily mediate the immune response to tumor antigens. As a result, the tumor site becomes a relative sanctuary in which immune cells and other host cellular components are significantly inhibited. Interestingly, the acidic pH of tumor microenvironment also appears to favor the recruitment of immunosuppressive cells, such as myeloid-derived suppressor cells, further promoting escape from immune surveillance, neo-angiogenesis and stromal remodeling [11]. In addition, there is evidence that an acidic extracellular pH promotes invasiveness and metastatic behavior in several tumor models [12,13], through a number of mechanisms such as increased traffic of acidic vesicles [14] and increased release of exosomes [15], whose pro-tumorigenic features are clearly emerging [16,17], lytic enzyme activation and matrix destruction [12,18] as well as aberrant phagocytic activity [19].

Clearly, these complex interactions of tumor and microenvironment represent a dynamic non-linear system and, as a result, a number of multidisciplinary groups at the interface between scientific fields, i.e. physics, mathematics and biology, are attempting to provide a unified frame to address these changes theoretically and experimentally [20-22]. Understanding the interactions of the physical microenvironment and cellular properties will be required to exploit the tumor metabolism as a therapeutic strategy.

One emerging theme from this work is that the detoxification mechanisms that allow cancer cells to survive in extremely hostile conditions represent very appealing therapeutic targets. Thus, inhibition of membrane-bound carbonic anhydrases, proton pumps and transporters, which appear to be major pathways used by tumor cells to export protons [23-30], may represent a novel and promising target for exerting a pleiotropic, multifaceted suppression of cancer cell growth and progression. Several lines of evidence suggest that the majority of human cancers may potentially be responsive to therapies based on inhibition of mechanisms underlying tumor acidification [25-27].

Indeed, recent research has supported the idea that inhibiting tumor proton pumps and transporters may deprive cancer cells of a key mechanism of detoxification [7,22,23]. During the last decade, this H^+^- related approach mainly focused on the dynamics of protons in cancer has integrated many basic and clinical aspects, as well as several areas of cancer research and treatment, under a unified perspective and paradigm in modern translational oncology [31]. However, to date proton pump inhibition as an antitumor strategy has remained neglected in the clinic, despite mounting evidence of its potential use as an inexpensive and relatively non-toxic treatment. For example, a class of proton pump inhibitors currently used to treat peptic ulcers has now entered clinical trials for treatment of patients affected by melanoma and osteosarcoma with the endpoints of evaluating the chemosensitization effect of pre-treatment with high dosages of esomeprazole.

Several research groups scattered all over the world have recently started to investigate various aspects of proton dynamics in cancer cells, with quite encouraging preliminary results. With the intent of unifying investigators involved in this line of research the First International Symposium entitled "Proton transport and its inhibition (PTI) in the etiopathogenesis, diagnosis and treatment of cancer" was held in Madrid, Spain, in April 2009. At this meeting we explored the main aspects of proton dynamics and the potential involvement in cancer etiology, etiopathogenesis and treatment. The main goals of this International Symposium were: a) To lead towards a unified and integrated understanding of the main role of H^+^ dynamics in modern cancer research; b) To discuss the more recent scientific data of intra and extracellular pH abnormalities in the onset of cancer as well as in its local and metastatic progression, focusing mainly on the molecular mechanisms driving the alterations of pH in various tumor types and tissues; c) To shed light on new potential targets for inducing selective apoptosis and other therapeutic interventions in malignant tumors and leukemias resistant to traditional treatments.

Both basic researchers and clinical oncologists participating at this meeting thought it was time to create a Society to attract the attention of lay people and the rest of the scientific community with respect to the emerging importance of acidity and proton dynamics in cancer pathogenesis and treatment. An intensive crosstalk between meeting attendees led to the formation of the "International Society for Proton Dynamics in Cancer" (ISPDC) in January 2010. This society is composed of researchers from all over the world who share the conviction that targeting tumor acidic pH could represent a significant advance in the treatment of most solid malignant tumors. Decreasing proton production, blocking proton extrusion, or increasing extracellular pH could result in a selective strategy to increase tumor cell death and/or to reduce proliferation and invasion.

The newly formed "International Society for Proton Dynamics in Cancer", focusing on the aspects of pH and/or dynamics of protons in cancer, will meet again during its First International Meeting in Rome on September 27th-28th 2010 http://ispdc.net. Both basic researchers and clinical oncologists are called upon to participate in this meeting where we hope to create an ideal environment to discuss several aspects of these new therapeutic approaches and develop implementation plans. The aim of the meeting is to foster translational research and stimulate interdisciplinary collaboration for developing new treatment targets and more specific and less toxic therapeutic strategies based on the most recent knowledge on proton dynamics in tumor cell biology.

## Competing interests

The authors declare that they have no competing interests.

## Authors' contributions

All authors gave intellectual contributions and participated in writing the manuscript. All authors read and approved the final manuscript.

## References

[B1] Nygren P Larsson R Overview of the clinical efficacy of investigational anticancer drugs J Intern Med 2003 253 46 75 10.1046/j.1365-2796.2003.01098.x 12588538

[B2] Gatenby RA A change of strategy in the war on cancer Nature 2009 459 508 509 10.1038/459508a 19478766

[B3] Hsu PP Sabatini DM Cancer cell metabolism: Warburg and beyond Cell 2008 134 703 707 10.1016/j.cell.2008.08.021 18775299

[B4] Pouyssegur J Dayan F Mazure N Hypoxia signaling in cancer and approaches to enforce tumor regression Nature 2006 441 437 443 10.1038/nature04871 16724055

[B5] Jin S DiPaola RS Mathew R White E Metabolic catastrophe as a means to cancer cell death J Cell Sci 2007 120 379 383 10.1242/jcs.03349 17251378 PMC2857576

[B6] Tennant DA Duran RV Gottlieb E Targeting metabolic transformation for cancer therapy Nat Rev Cancer 2010 10 267 277 10.1038/nrc2817 20300106

[B7] Fais S De Milito A You H Qin W Targeting vacuolar H^+^-ATPases as a new strategy against cancer Cancer Res 2007 67 10627 10630 10.1158/0008-5472.CAN-07-1805 18006801

[B8] Cardone RA Casavola V Reshkin SJ The role of disturbed pH dynamics and the Na^+^/H^+^ exchanger in metastasis Nat Rev Cancer 2005 5 786 795 10.1038/nrc1713 16175178

[B9] Supuran CT Carbonic anhydrases: novel therapeutic applications for inhibitors and activators Nat Rev Drug Discov 2008 7 168 181 10.1038/nrd2467 18167490

[B10] Kenan DJ Wahl ML Ectopic localization of mitochondrial ATP synthase: a target for anti-angiogenesis intervention? J Bioenerg Biomembr 2005 37 461 465 10.1007/s10863-005-9492-x 16691484

[B11] Gabrilovich DI Nagaraj S Myeloid-derived suppressor cells as regulators of the immune system Nat Rev Immunol 2009 9 162 174 10.1038/nri2506 19197294 PMC2828349

[B12] Martinez-Zaguilan R Seftor EA Seftor RE Chu YW Gillies RJ Hendrix MJ Acidic pH enhances the invasive behavior of human melanoma cells Clin Exp Metastasis 1996 14 176 186 10.1007/BF00121214 8605731

[B13] Gatenby RA Gillies RJ A microenvironmental model of carcinogenesis Nat Rev Cancer 2008 8 56 61 10.1038/nrc2255 18059462

[B14] Raghunand N Martinez-Zaguilan R Wright SH Gillies RJ pH and drug resistance. II. Turnover of acidic vesicles and resistance to weakly basic chemotherapeutic drugs Biochem Pharmacol 1999 57 1047 1058 10.1016/S0006-2952(99)00021-0 10796075

[B15] Parolini I Federici C Raggi C Lugini L Palleschi S De Milito A Coscia C Iessi E Logozzi M Molinari A Colone M Tatti M Sargiacomo M Fais S Microenvironmental pH is a key factor for exosome traffic in tumor cells J Biol Chem 2009 284 34211 34222 10.1074/jbc.M109.041152 19801663 PMC2797191

[B16] Iero M Valenti R Huber V Filipazzi P Parmiani G Fais S Rivoltini L Tumour-released exosomes and their implications in cancer immunity Cell Death Differ 2008 15 80 88 10.1038/sj.cdd.4402237 17932500

[B17] Al-Nedawi K Meehan B Rak J Microvesicles: messengers and mediators of tumor progression Cell Cycle 2009 8 2014 2018 19535896 10.4161/cc.8.13.8988

[B18] Lu X Qin W Li J Tan N Pan D Zhang H Xie L Yao G Shu H Yao M Wan D Gu J Yang S The growth and metastasis of human hepatocellular carcinoma xenografts are inhibited by small interfering RNA targeting to the subunit ATP6L of proton pump Cancer Res 2005 65 6843 6849 10.1158/0008-5472.CAN-04-3822 16061667

[B19] Lugini L Matarrese P Tinari A Lozupone F Federici C Iessi E Gentile M Luciani F Parmiani G Rivoltini L Malorni W Fais S Cannibalism of live lymphocytes by human metastatic but not primary melanoma cells Cancer Res 2006 66 3629 3638 10.1158/0008-5472.CAN-05-3204 16585188

[B20] Rauch C Toward a mechanical control of drug delivery. On the relationship between Lipinski's 2nd rule and cytosolic pH changes in doxorubicin resistance levels in cancer cells: a comparison to published data Eur Biophys J 2009 38 829 846 10.1007/s00249-009-0429-x 19296096

[B21] Rauch C On the relationship between drug's size, cell membrane mechanical properties and high levels of multi drug resistance: a comparison to published data Eur Biophys J 2009 38 537 546 10.1007/s00249-008-0385-x 19066880

[B22] Rauch C Pluen A Multi drug resistance-dependent "vacuum cleaner" functionality potentially driven by the interactions between endocytosis, drug size and Pgp-like transporters surface density Eur Biophys J 2007 36 121 131 10.1007/s00249-006-0113-3 17211622

[B23] Panagiotopoulou V Richardson G Jensen OE Rauch C On a biophysical and mathematical model of Pgp-mediated multidrug resistance: understanding the "space-time" dimension of MDR Eur Biophys J 2010 39 201 211 10.1007/s00249-009-0555-5 19888571

[B24] Lozupone F Perdicchio M Brambilla D Borghi M Meschini S Barca S Marino ML Logozzi M Federici C Iessi E de Milito A Fais S The human homologue of Dictyostelium discoideum phg1A is expressed by human metastatic melanoma cells EMBO Rep 2009 10 1348 1354 10.1038/embor.2009.236 19893578 PMC2799214

[B25] Wahl ML Owen JA Burd R Herlands RA Nogami SS Rodeck U Berd D Leeper DB Owen CS Regulation of intracellular pH in human melanoma: potential therapeutic implications Mol Cancer Ther 2002 1 617 628 12479222

[B26] Harguindey S Arranz JL Wahl ML Orive G Reshkin SJ Proton transport inhibitors as potentially selective anticancer drugs Anticancer Res 2009 29 2127 2136 19528473

[B27] Chiche J Ilc K Laferriére J Trottier E Dayan F Mazure NM Brahimi-Horn MC Pouysségur J Hypoxia-inducible carbonic anhydrase IX and XII promote tumor cell growth by counteracting acidosis through the regulation of the intracellular pH Cancer Res 2009 69 358 368 10.1158/0008-5472.CAN-08-2470 19118021

[B28] De Milito A Canese R Marino ML Borghi M Iero M Villa A Venturi G Lozupone F Iessi E Logozzi M Della Mina P Santinami M Rodolfo M Podo F Rivoltini L Fais S pH-dependent antitumor activity of proton pump inhibitors against human melanoma is mediated by inhibition of tumor acidity Int J Cancer 2010 127 207 219 19876915 10.1002/ijc.25009

[B29] De Milito A Fais S Tumor Acidity, Chemoresistance and Proton Pump Inhibitors Future Oncology 2005 1 779 786 10.2217/14796694.1.6.779 16556057

[B30] Izumi H Torigoe T Ishiguchi H Uramoto H Yoshida Y Tanabe M Ise T Murakami T Yoshida T Nomoto M Kohno K Cellular pH regulators: potentially promising molecular targets for cancer chemotherapy Cancer Treat Rev 2003 29 541 549 10.1016/S0305-7372(03)00106-3 14585264

[B31] Harguindey S Orive G Luis Pedraz J Paradiso A Reshkin SJ The role of pH dynamics and the Na^+^/H^+ ^antiporter in the etiopathogenesis and treatment of cancer. Two faces of the same coin-one single nature Bioch Biopyhs Acta 2005 1756 1 24 10.1016/j.bbcan.2005.06.004 16099110

